# Scanning electron microscopy and extended viability testing as a tool to evaluate the safety of MALDI-TOF extracts for risk group 3 spore-forming bacteria

**DOI:** 10.1099/jmm.0.001992

**Published:** 2025-07-15

**Authors:** Kym S. Antonation, Britni L. Baron, Timothy F. Booth, Daniel R. Beniac, Cindi R. Corbett

**Affiliations:** 1 National Microbiology Laboratory, Public Health Agency of Canada, Winnipeg, Manitoba, R3E 3R2, Canada

**Keywords:** biosafety, bio-threat bacteria, inactivation, MALDI-TOF MS, viability testing

## Abstract

**Introduction.** Matrix-assisted laser desorption/ionization-time of flight (MALDI-TOF) MS for rapid identification of risk group 3 (RG3) bacteria is impeded by the following two main limitations: (a) equipment and maintenance costs for instruments placed within containment and (b) lack of a validated inactivation protocol to move RG3 material to a lower containment level. A validated inactivation method would improve operations of public health laboratories by allowing safe triage of potential RG3 agents. Albeit a validated, zero-risk inactivation protocol is unlikely, scientific interrogation of methods to identify and mitigate procedural biosafety risks is vital for institutional risk assessment.

**Gap.** To investigate the effect of a standard MALDI-TOF chemical extraction, hypothesized to alter cells, allowing passage through a filter and maintaining ability to replicate, this study paired visualization using a scanning electron microscope (SEM) with extended viability testing.

**Aim.** This work is intended to support risk assessments for the removal of material from a containment laboratory for MALDI-TOF MS.

**Methodology.** A standard set of *Bacillus cereus* and *Bacillus anthracis* vegetative and spore preparations was treated with a formic acid:acetonitrile extraction, with or without filtration, and plated on five types of media to monitor growth over 14 days. SEM images were taken of treated and untreated preparations, prior, during and after filtration across two filters. Reference beads provided accurate pore size measurements.

**Results.** SEM demonstrated no difference in treated and untreated cells but did indicate the ineffectiveness of cellulose filters compared to PVDF filters. Growth was observed in preparations that did not include PVDF filtration, whereas all preparations (*n*=60) that included PVDF filtration were 100% non-viable. Although non-viability was observed, an important finding was the passage of 0.262 and 0.173 µm microspheres through the 0.1 µm PVDF filter. Growth of unfiltered preparations was detected between 1 and 7 days.

**Conclusions.** This investigation demonstrates the value of interrogating materials used for bacterial inactivation, highlighting significant issues in the application of filters for exclusion purposes. Visual examination via SEM was key to providing evidence towards a low-risk inactivation method. These findings, with an understanding of limitations identified herein, can be used to inform risk assessments for the removal of RG3 bacteria from containment.

**Impact Statement**

There are limited data or systematic studies on the effect of chemical action on spore-forming bacterial cells and/or filters used during the preparation of MALDI-TOF MS extracts that could lead to the escape of viable spores. It has been suggested that, perhaps, the application of chemicals such as formic acid and acetonitrile can cause physical changes to the spore structure, allowing passage through the filter. In this study, we demonstrate that this is unlikely and provide a body of work that provides science-based evidence for containment laboratory biosafety risk assessment(s).

## Introduction

Matrix-assisted laser desorption/ionization-time of flight (MALDI-TOF) MS for rapid bacterial identification is firmly ensconced as an integral part of a diagnostic microbiology laboratory workflow. As such, a large body of work has been amassed with respect to protocol development and advancement in the use of the technology [1]. Despite goals within the community of a safe, universal method, particularly for intact cell identification methods, optimal sample preparation is challenging, and a universal method for the inactivation of high-consequence bacterial pathogens has limited studies of rigour to support evidence-based decisions for institutional biosafety practices [2,3]. It is not financially feasible for most microbiology laboratories to have a dedicated mass spectrometer in a containment level 3. As such, a body of successive orthogonal studies demonstrating the concordance for bacterial inactivation is requisite for well-informed risk assessments required for the removal of material(s) from a containment laboratory.

Historically, published reports related to inactivation methods for risk group 3 (RG3) spore-formers (i.e. *Bacillus anthracis*) have yielded diverse findings, both in agreement and in conflict towards this aim [4,8]. The unfortunate release of viable *B. anthracis* material in 2014 from the Centre for Disease Control and Prevention (CDC) [9] initiated several studies that promoted the multistep process of chemical extraction by the formic acid:acetonitrile method, the requirement for centrifugation and filtration to obviate remaining cells and/or spores, and finally the need for extended viability testing with appropriate media [4,8, 10]. Lasch and Weller [4,6, 7] have published reports evaluating filter material and efficacy, demonstrating the need for chemical (PVDF) filter material rather than organic, cellulose filters that can disintegrate with the application of MALDI-TOF chemical extraction reagents.

Nonetheless, breakthrough growth of spores has been observed despite chemical extraction and appropriate filtration [7]. Several theories have been postulated around this, notably the idea that cells and/or spores are ‘altered’ in size or shape by the application of the chemicals used for extraction, allowing them to pass through a filter. *B. anthracis* spores have been measured with a mean diameter of >0.8–<0.9 µm and a mean length of 1.26–1.67 µm [7] and, thus, should theoretically not pass through a 0.1 µm filter. Filter composition and structure could be the root cause of breakthrough growth, or alternatively, the method used to detect breakthrough growth may have been suboptimal (liquid versus solid media, inoculum size or others) [2,8, 11]. Thus, the intent of this study was to evaluate these questions through the application of scanning electron microscopy (SEM) and extended viability testing protocols to a set of standardized treated and untreated preparations of *B. anthracis* and its surrogate as a simple way to contribute to the growing body of evidence towards a standard, effective method for viability testing.

## Methods

### Sample preparation

This study was split into two phases: (1) SEM visualization and (2) extended viability testing. For the SEM visualization within a containment level 2 environment, *B. cereus* (ATCC 14579) was used as a surrogate. For the extended viability testing, three different strains of *B. anthracis* (Sterne, Ames and 17T5) were used to represent a spectrum of strains. All preparations were done in duplicate. For all isolates, a 1 McFarland standardized concentration of vegetative cells and spores, correlating to 10^8^–10^9^ c.f.u. ml^−1^ as determined by agar colony counts and digital PCR, was prepared. Vegetative cells were prepared by culturing the isolates on SBA and incubating for 16–24 h at 37 °C/5% CO_2_. Spores were prepared by the inoculation of bacteria in 1/10 Columbia broth containing 0.1 mM MnSO_4_ at 37 °C shaking for 96 h. Culture was centrifuged at 4,900 x g for 15 min with the supernatant discarded, followed by three washes with sterile deionized water and a final resuspension in sterile deionized water. Bacterial suspension was heat inactivated at 80 °C for 10 min to kill vegetative cells.

The vegetative cells and spore preparations were chemically extracted in a microfuge tube using 100% ethanol, 70% formic acid and 100% acetonitrile as described previously (Tracz JCM 2015). Briefly, 900 µl 100% ethanol was added to the test samples, mixed by inversion and centrifuged at 14,000 x g for 3 min to pellet cells/spores. Ethanol was removed completely and pellets were left to dry for 5 min. Pellets were re-suspended in 60 µl of 70% formic acid and incubated for 5 min at room temperature. A total of 60 µl of 100% acetonitrile was added to the suspension and mixed well. The tubes were centrifuged at 14,100 x g for 2 min to create a pellet. For the samples undergoing filtration, 60 µl of supernatant was added to the microfuge filter in question (cellulose or PVDF) and centrifuged at 8,700 x g for 3 min.

**Fig. 1.**
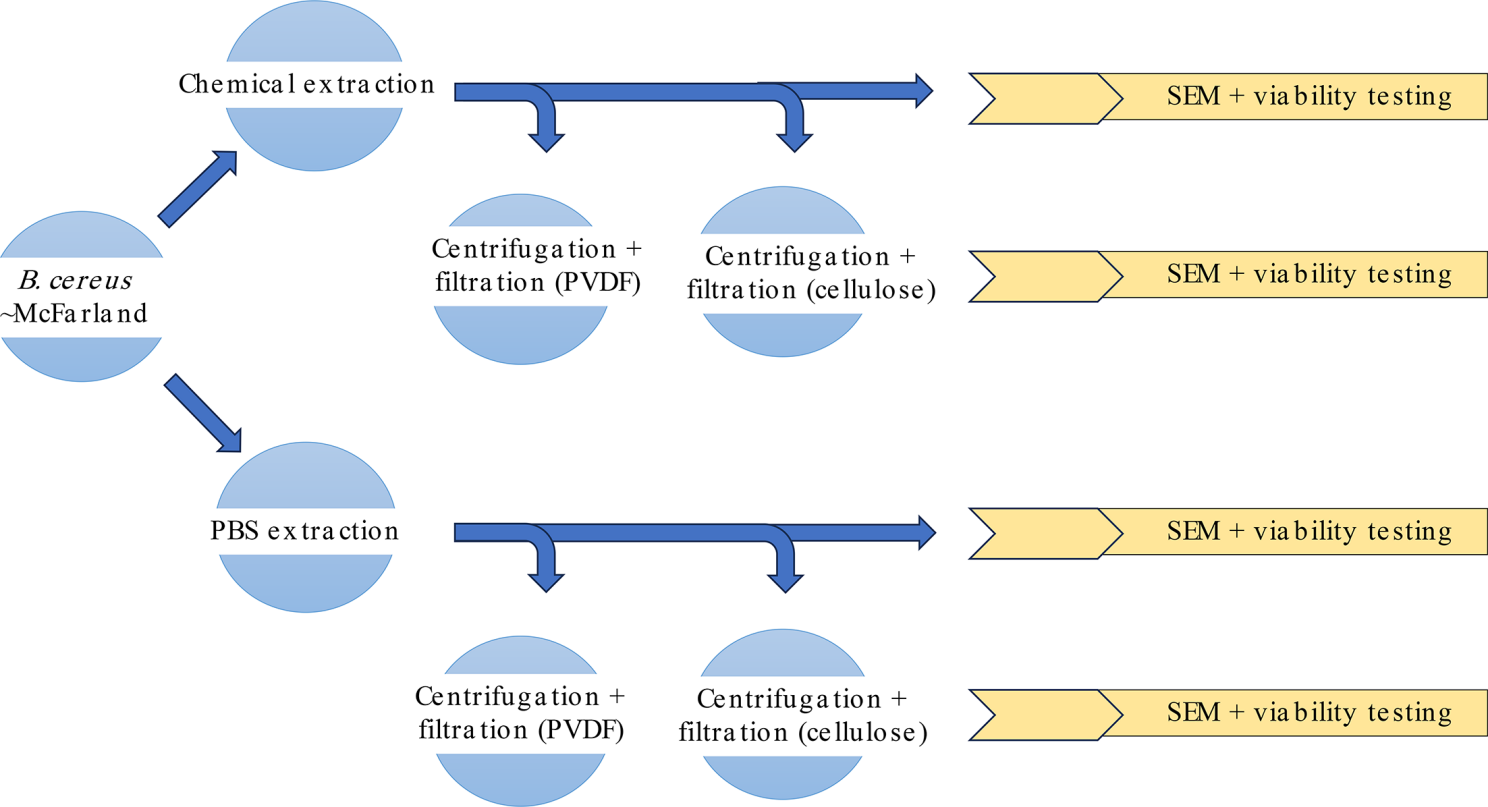
Sample flow for SEM visualization of surrogate *B. cereus* vegetative and spore preparations. Standardized sample material was viewed with and without chemical treatment, before and after filtration with two filter materials. Material was deposited on an EM collection filter (SPI–Pore polycarbonate track etch filter) for visualization.

A portion of the standardized cells/spores was left untreated as a control. For SEM visualization, treated and untreated (in Phosphate Buffered Saline (PBS)) *B. cereus* cells/spores were sampled at different points for SEM as per Fig. 1. Untreated and chemically treated samples (60 µl) were directly observed via SEM prior to filtration to observe for assessment of physical changes due to chemical treatment, as were the sample filtrates (45 µl) and physical filters from either a 0.1 µm pore size PVDF microcentrifuge filtration unit (EMD Millipore, Billerica, Massachusetts, USA) or a 0.2 µm pore size regenerated cellulose filter (Chrom Tech, Apple Valley, MN, USA) (Fig. 1).

For initial viability assessment, 45–60 µl or 50% of each sample was inoculated into 7.8 ml MGIT^©^ with supplement, incubated at 37 °C in a BACTEC MGIT^©^ 960 for 14 days, plated on SBA, 37 °C/5% CO_2_, and then monitored for 14 days to check for viable cells.

For extended viability testing, treated and untreated *B. anthracis* cells/spores were treated as above. However, in this phase, only 0.1 µm pore size PVDF filters were applied, and 120 µl of each sample was inoculated to Tryptic Soy Broth (TSB), Brain Heart Infusion (BHI) broth, MGIT^©^ broth with supplement, Sheep Blood Agar (SBA) and BHI agar plates. TSB and BHI broths were plated daily (200 µl) to SBA to detect viable colonies for 14 days. MGIT^©^ broth was machine-monitored for 14 days, followed by plating to SBA to confirm no growth. All growth was confirmed as *B. anthracis* via an in-house three-target quantitative, real-time PCR assay and a canonical SNP assay to confirm correct strain.

### Electron microscopy

All samples prepared were filtered and viewed using 13 mm diameter SPI-Pore Polycarbonate Track Etch Filters with 100 nm pores (SPI supplies, West Chester, Pennsylvania, USA), held in 13 mm Swinnex^®^ filter holders (EMD Millipore, Billerica, Massachusetts, USA). These filter units were attached only to syringes with Luer–Lok^®^ attachments to ensure that the syringe and filter holders would remain coupled so that no sample leaks would occur. All syringe filtering was performed using a Legato 200 syringe pump (kd Scientific, Holliston, Massachusetts, USA) operated with a flow rate of 1 ml/min to ensure reproducible filtration conditions. The subsequent filter preparation of all the specimens was done in a biological safety cabinet, using Luer–Lok^®^ syringes to pass all fluids through the filter assembly. The steps used were as follows: using a 2 ml syringe, 2 ml of PBS was passed through the apparatus to wet the filter. Next, 0.2 ml of the extract suspension is applied to the filter using a 1 ml syringe, followed by a 5 ml wash with PBS using a 5 ml syringe. Then using 2 ml syringes, the filter was washed with 2 ml 50% ethanol, 2 ml 70% ethanol, 2 ml 85% ethanol, 2 ml 95% ethanol and 2 ml 100% ethanol. The filter apparatus was then disassembled, and the filter was allowed to air dry.

Once dry, the filters were cut or directly mounted onto an SEM stub. First, a double-sided adhesive carbon disc is stuck to the metal stub, and then the filter is stuck to the carbon disc, bacteria side up. Silver flash paint was then used to create a conductive contact between the stub and the filter paper. The samples were then sputter coated with gold using a Quorum Q150R S sputter coater (Quorum Technologies, Lewes, United Kingdom).

Specimens were imaged in an SEM (JEOL CarryScope, JEOL Ltd., Tokyo, Japan) operated at 6 kV, with a spot size of 20, a 9 mm working distance and at nominal instrument magnifications ranging from X100 to X5,000. Digital images were acquired using the secondary electron detector. Measurements were made using the Image J software package [12] using the straight line or freehand line tool and the analysed/measured function.

### Microsphere preparation for scanning electron microscopy

Standard measurement spheres of varying sizes – 1.03, 0.5, 0.262 and 0.173 µm (Canemco, Lakefield, QC, Canada, and SPI supplies, West Chester, Pennsylvania, USA) – were utilized to measure centrifugal filter size exclusion. For these investigations, the 0.1 µm pore size microcentrifuge filtration unit (EMD Millipore, Billerica, Massachusetts, USA) and 0.2 µm pore size regenerated cellulose filter (Chrom Tech, Apple Valley, MN, USA) units were tested. The protocols used were identical to those described above. Briefly, the microspheres were first processed by centrifugal filtration, and the filtrate was subsequently filtered using SPI-Pore Polycarbonate Track Etch Filters with 100 nm pores, which would retain any microspheres that pass through the centrifugal filters.

## Results

### Scanning electron microscopy

**Fig. 2.**
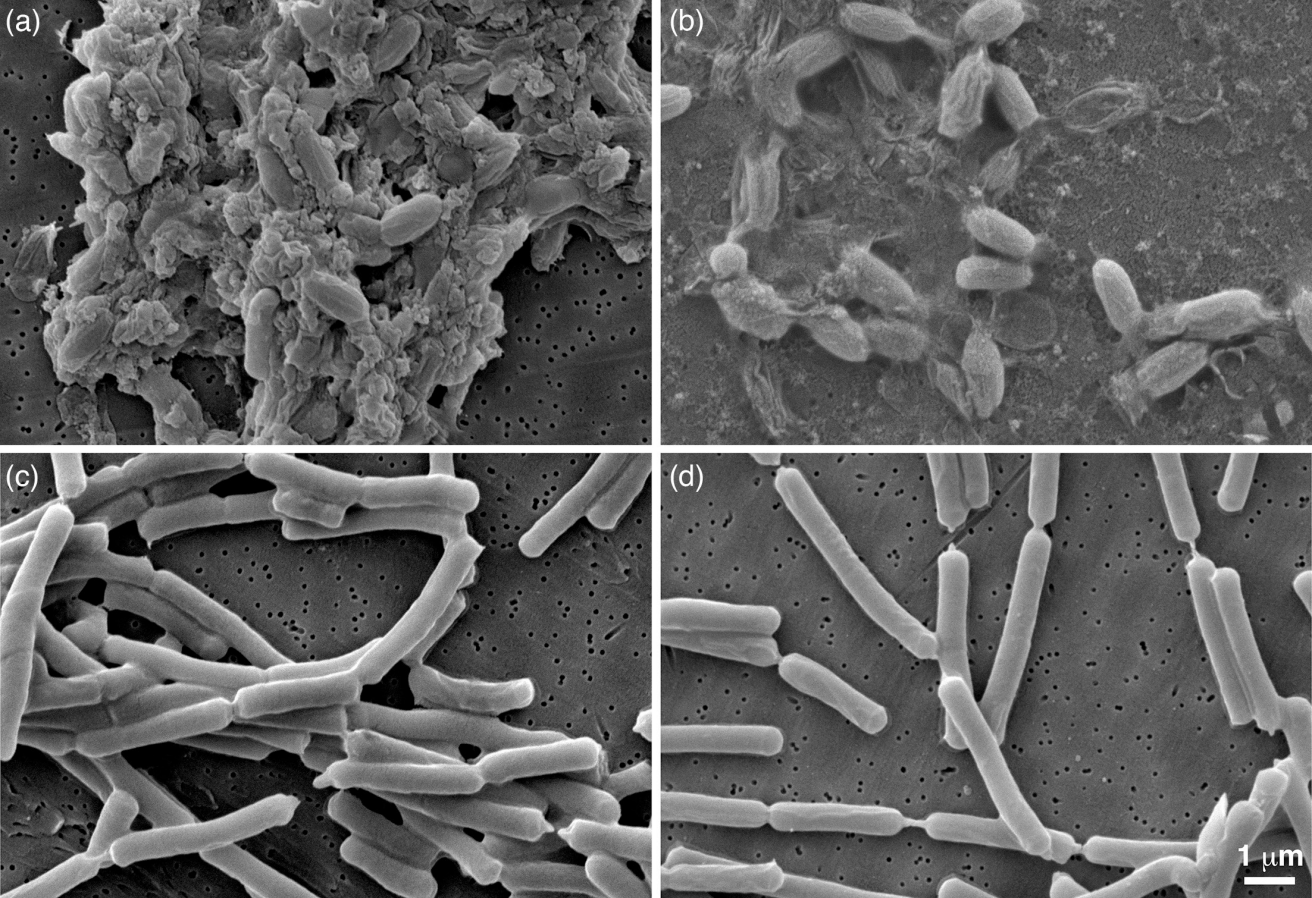
SEM of untreated, control spores (**a**) and vegetative cells (**c**) against acetonitrile and formic-acid-treated spores (**b**) and treated vegetative cells (**d**) of *B. cereus*, deposited on the EM collection filter (SPI-Pore Polycarbonate Track Etch Filter) for visualization.

Upon examination of the cells and spore preparations pre- and post-treatment, no difference in size was observed; the treatment did not appear to cause the cells to ‘shrink’, allowing them to be able to pass through a filter (Fig. 2). Untreated spores measured 1.496±0.137 by 0.672±0.044 (*n=*39) compared with treated spores at 1.491±0.130 by 0.670±0.046 (*n=*42). The treated spores did appear less aggregated than the untreated.

**Fig. 3.**
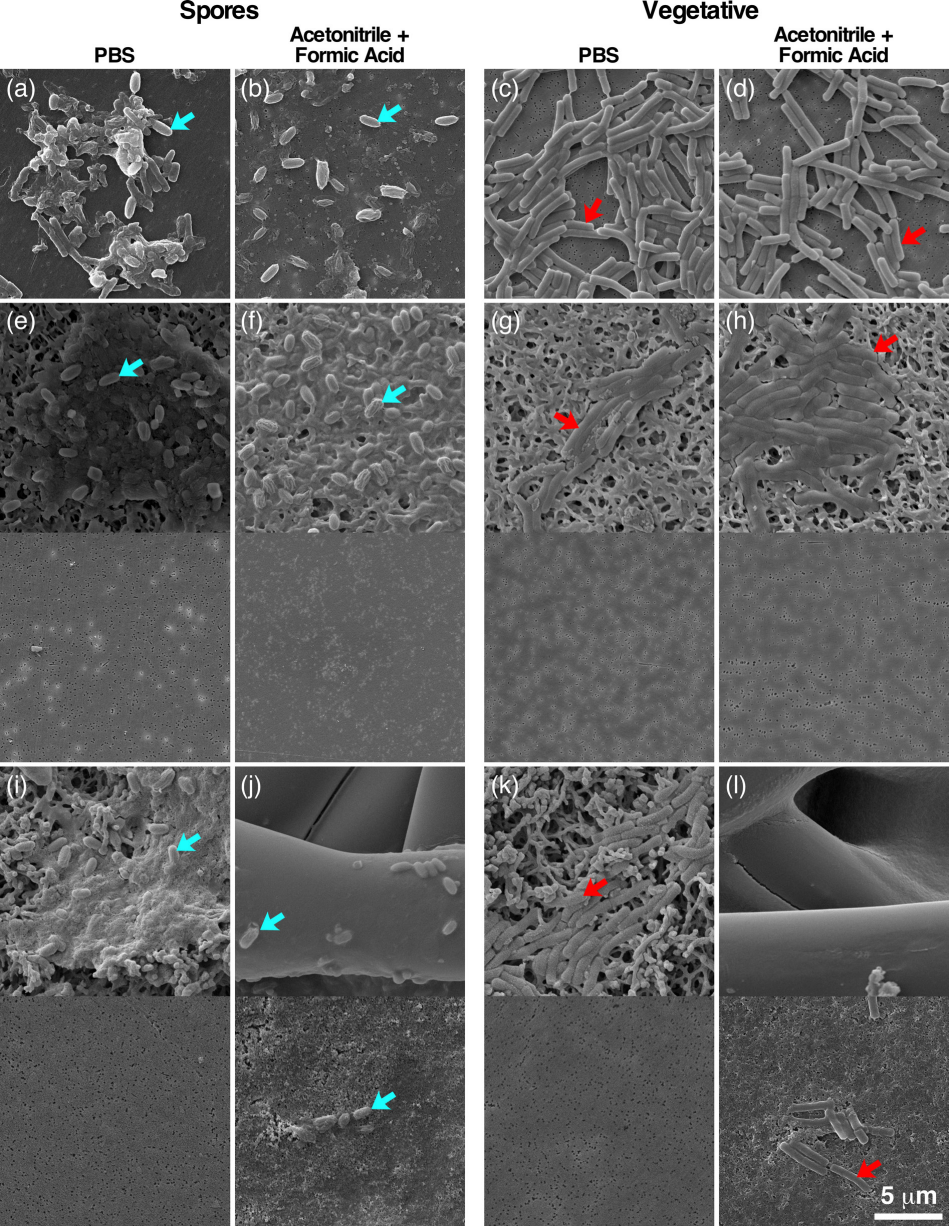
Filtration of spores and vegetative cells. (**a-d**) Unfiltered spores and vegetative *B. cereus* cells were treated with either PBS or acetonitrile and formic acid. (**e-l**) Image pairs showing the cells deposited on the filter (top), and the flow through filtrate which was subsequently captured on a SPI-Pore filter membrane (bottom). PVDF Millipore (**e-h**) and cellulose Chrom Tech filters (**i-l**) were tested. Spores and vegetative cells are denoted with blue and red arrows, respectively.

**Fig. 4.**
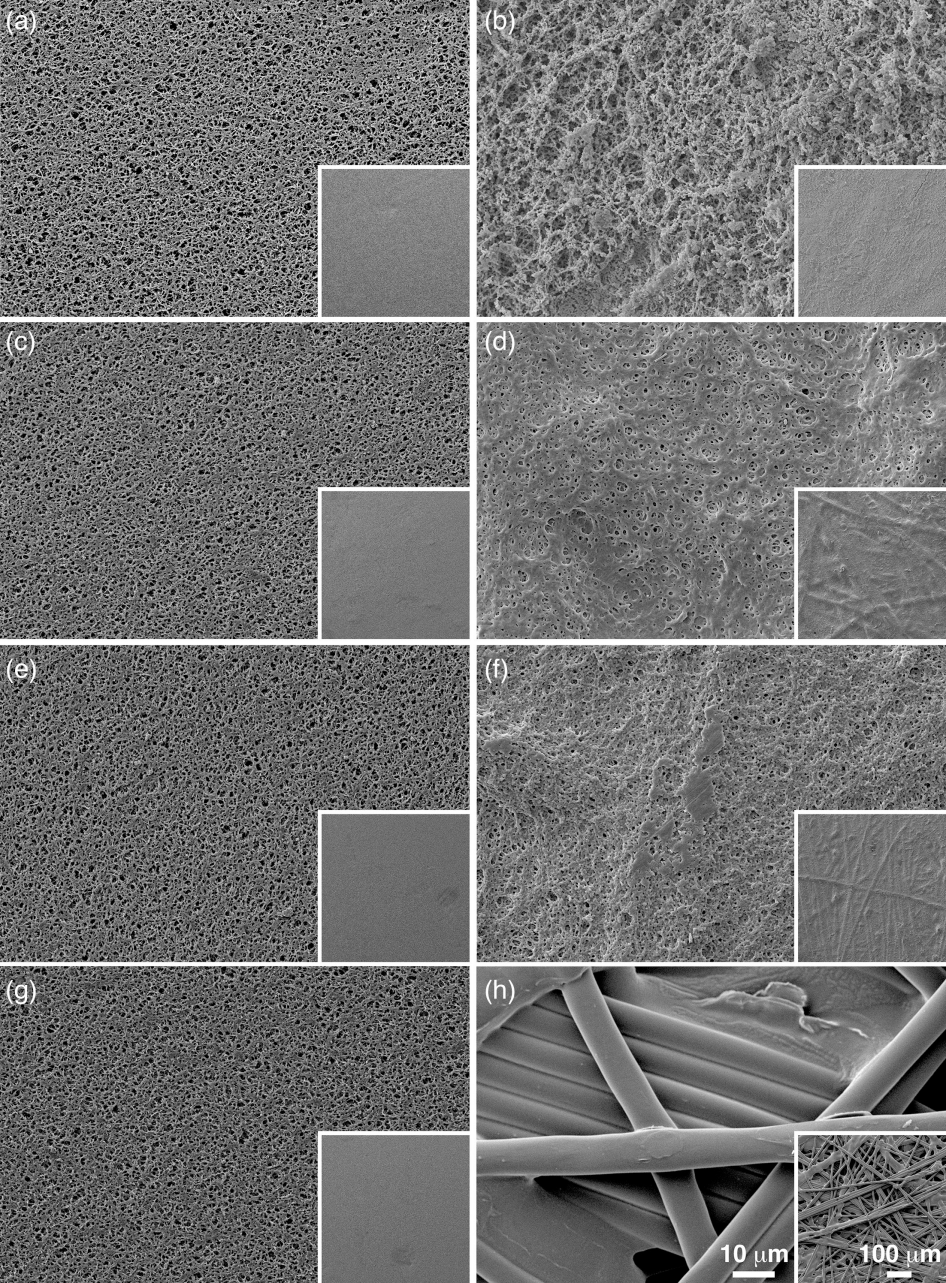
Chemical treatment of filter membranes. PVDF Millipore (**a,c,e,g**), and cellulose Chrom Tech filters (**b,d,f,h**) were treated with the chemicals used to inactivate *B. cereus* for MALDI-TOF. The filters were treated with: no chemicals (**a,b**), acetonitrile (**c,d**), formic acid (**e,f**) and acetonitrile and formic acid (**g,h**). Each image has an inset in the lower right corner at lower magnification.

Replicates of vegetative and spore preparations filtered using the PVDF filters demonstrated that no cells or spores passed through the membrane (Fig. 3e–h). This was supported by no growth on all media used throughout this study. Additionally, the treatment had no observable effect on the PVDF filter membrane integrity (Fig. 4a, c, e and g). In contrast, the same preparations filtered using regenerated cellulose filters did show passage of cells and spores through the filter matrix (Fig. 3i–l) and did exhibit growth identified as *B. cereus*. The treatment of the regenerated cellulose filters with acetonitrile, formic acid or both chemicals resulted in the degradation of the membrane (Fig. 4b, d, f and h). In this case, the combination of both chemicals caused the most degradation of the filter, which in turn permitted passage of spores and vegetative cells through what was left of the filter membrane (Fig. 3j and l).

**Fig. 5.**
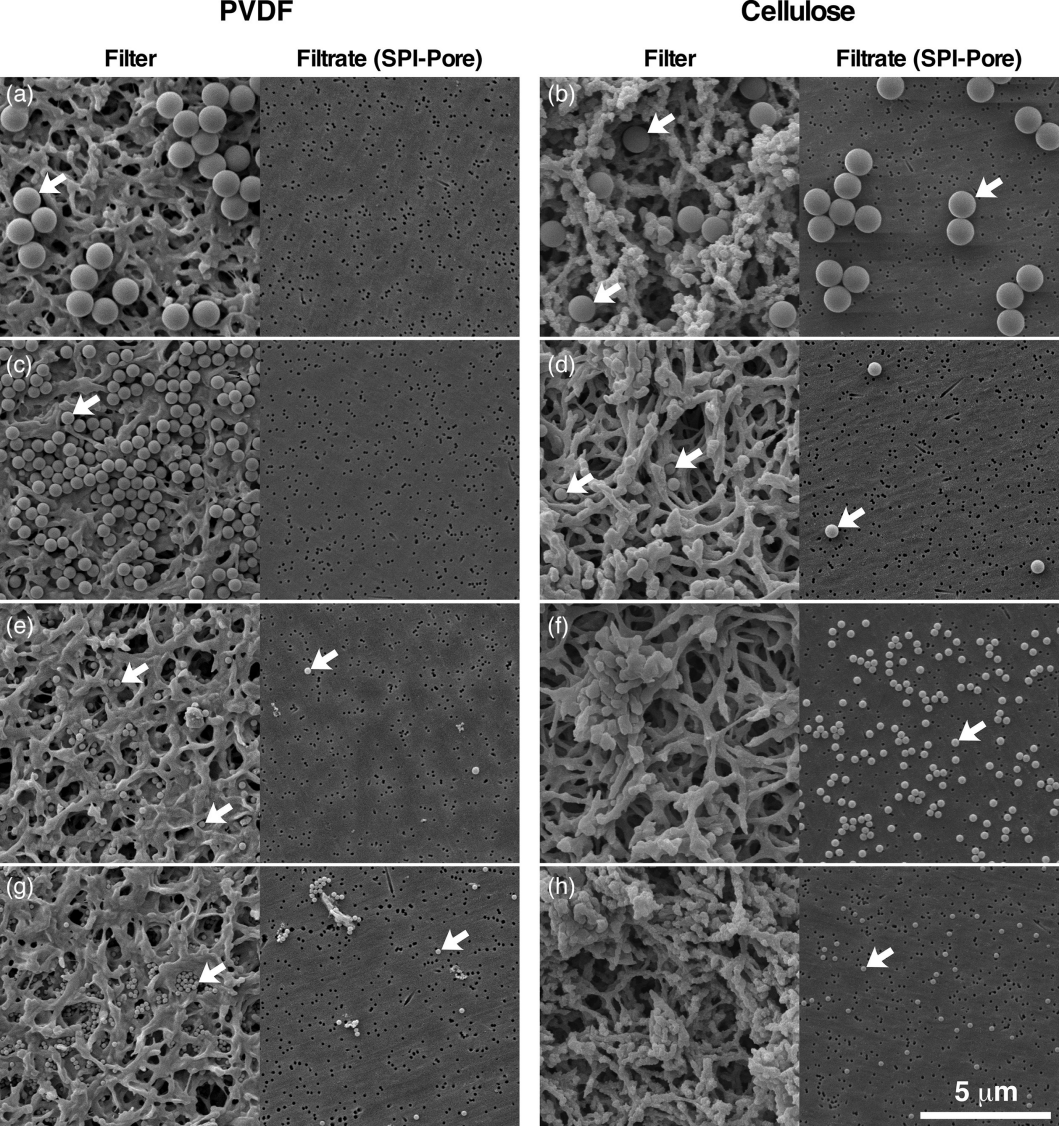
Test of size exclusion of filter membranes. Microspheres were used to test 0.1 µm pore PVDF Millipore (**a,c,e,g**), and 0.2 µm pore cellulose Chrom Tech filters (**b,d,f,h**). The filters were tested with the following sphere sizes: 1.03 µm (**a,b**), 0.5 µm (**c,d**), 0.262 µm (**e,f**) and 0.173 µm (**g,h**). For each test, a pair of SEM images is presented; one is of the surface of the filter (left), and the second is the filtrate which was deposited on a SPI-Pore membrane that would not permit further passage of the spheres (right). Individual spheres are denoted with white arrows.

Standard-sized microspheres demonstrated that the filters used differ in the ability for size exclusion (Fig. 5). The 0.2 µm cellulose filters allowed the passage of all test spheres, whereas the 0.1 µm Millipore PVDF filters allowed passage of the 0.262 µm and 0.173 µm micron spheres, excluding the 0.5 µm and 1.03 µm spheres. While the average size might be more or less accurately measured as 0.1 µm, clearly there are much larger pores throughout the layered matrix as shown. Only the SPI-Pore filters used to collect the filtrate had a physical pore size matching the 100 nm (0.1 µm) pore size that the product was quoted to have.

Centrifugation of the extraction before centrifugal filtration was found necessary; otherwise, the extract had an abundance of intact cells that were observed to overload the filters and could possibly cause them to crack or push cells through the matrix (image not shown).

### Viability

Within the initial phase of viability testing, the growth of *B. cereus* was observed in several populations, notably, in treated spores prior to filtration, untreated spores filtered through the cellulose filter, as well as treated vegetative cells filtered through the cellulose filter. This indicated that (a) the chemical treatment is not effective at fully inactivating spore or vegetative cells as observed in prior work [10,13] and (b) the cellulose filters were not size exclusionary to agents >0.2 µm, correlated by SEM. In contrast, all samples passed through the PVDF filter were negative upon viability testing.

Due to the above results, a second phase of this study utilized a larger volume for viability testing (120 µl, 100%) and extended testing to five types of media. All PVDF filtrate samples (*n*=60) were negative for growth. All samples that were not filtered (chemically treated or native) had observable growth on SBA, BHIA, TSB and BHI media (Table 1). Vegetative cells had observable growth (1–2 colonies) on solid media (BHIA and SBA) between 2 and 7 days. The broth media, of which 200 µl was sub-cultured to solid media after 24 h incubation, detected viable cells; however, the visualization on solid media took 96 h incubation. For the unfiltered spore extracts, observable growth was detected on all media types (except MGIT) within 1 day on solid media, and between 24 and 96 h after subculture from liquid media. The unfiltered spores were detected 100% of the time using BHI solid and liquid media, as well as TSB. SBA direct plating yielded positive detection in 66% of samples. MGIT^©^ was unsuccessful in detecting viable cells within the BACTEC system; no growth was detected at any time by the machine. Suspecting this was related to chemical inhibition impeding fluorescence detection, MGIT tubes inoculated with unfiltered spore extracts were removed from the instrument at day 14, and the entire broth contents were plated to three large SBA plates (~2.8 ml each). All plates showed lawn growth of *B. anthracis*, verified by qPCR.

**Table 1.** Growth detection of chemically treated, unfiltered *B. anthracis* vegetative cell and spore replicates on solid (BHIA, SBA) and liquid (TSB, BHI) media

		Days to detection by media type(replicate 1/replicate 2)			
	Strain	SBA	BHIA	TSB	BHI broth
Spores	Sterne	- / 1	1/1	2/2	2/2
	Ames	1/1	1 / 1	2/2	2/2
	17T5	- / 1	1/1	5/5	5/5
Vegetative	Sterne	- / 5	2/5	- / -	5 / -
	Ames	- / -	- / -	- / -	- / -
	17T5	5/2	5/7	5/5	5/5

This time includes plating of liquid media after 24 h to SBA for visual detection (5 days=24 h in liquid media and 96 on solid subculture). A ‘-’ indicates no growth observed.

## Discussion

A single biosafety incident with a high-consequence pathogen can have deleterious effects on both the individuals and institutions involved. To mitigate such events, a solid basis of scientific evidence related to the pathogen of interest and its use is requisite towards an informed and balanced risk assessment. In this study, we sought to answer unresolved questions surrounding the safe handling and movement of a *B. anthracis* extract to a lower containment area for MALDI-TOF identification.

Research examining the safe handling and inactivation of high-consequence bacteria for MALDI-TOF analysis, while preserving the integrity of the spectral profiles, has been accumulating for >20 years; indeed, Lasch *et al*. [4] undertook a novel study in 2008 where spore formers (loads up to 10^10^) were subjected to trifluoroacetic acid (TFA) extraction, centrifugation and filtration that resulted in 100% sterility with adequate spectral profiles. This work used transmission electron microscopy to examine the effect of the TFA extraction and demonstrated evidence that the TFA did cause morphological changes through protein solubilization to the spores exposed. Despite this, the TFA extraction alone reduced the spore count by ~six logs, and centrifugation and filtration (0.22 µm) were recommended to obviate any remaining spores. Filters of 0.1 µm in size were recommended by Dauphin and Bowen in 2009 [14] for filtration of *B. anthracis* samples, which was supported in 2012 by Drevinek *et al*. [13] and others, who suggested a filtration step for highly virulent pathogens, since the inactivation of *B. anthracis* spores could not be guaranteed with MALDI-TOF chemical extraction alone [4,8]. This was readily observed within this study as well, with viable cells detected in almost all unfiltered extracts treated with formic acid and acetonitrile. It is, therefore, obvious that an appropriate exclusion mechanism must be applied to render the extracts non-viable.

In 2014, after the CDC reported a biosafety incident involving the removal of viable *B. anthracis* organisms from containment after processing for MALDI analysis, there were calls to action for study into the chemical action on filter membranes and its effect on bacterial cells and spores, to identify potential structural changes or ‘shrinkage’ that may be leading to escape mechanisms [2,7]. Effectively, the work achieved herein supports both previously generated research and physically illustrates that the application of standard chemicals used for MALDI-TOF formic acid:acetonitrile extractions does not cause ‘shrinkage’ of cells or spores as hypothesized in the community (Fig. 2), which would allow potential passage through a filter matrix. Moreover, it provides physical evidence of the effectiveness of different filter materials and the need to be cognizant of this.

Spores have been measured with mean dimensions of >0.8–<0.9 µm × 1.26–1.67 µm [7]. In this study, using reference beads of 1.03, 0.5, 0.262 and 0.173 µm, we observed the passage of all beads through 0.2 µm regenerated cellulose filters, and the passage of the 0.173 and 0.262 µm spheres with the 0.1 µm PVDF filter. This leads us to believe that the pore measurements given are not exact, but rather intended to be size exclusionary. Furthermore, the application of the treated extracts to the cellulose filters showed evidence of disintegration (Fig. 4); subsequently, viable cells were recovered from these extracts. This is in direct agreement to observations of viable cells post-extraction using cellulose filters employed by Weller *et al*. [6], and it confirms the hypothesis of chemical action on the filters themselves and inconsistencies in pore sizes. Images shown here demonstrate that the pores are not exact, which explains the passage of slightly larger spheres through the matrix under centrifugal force.

In our hands, PVDF filters did not permit passage of any reference spheres >0.5 µm, theoretically excluding standard bacillus spores from passage. Indeed, all 60 samples extracted in phase 2 of this work and filtered using a 0.1 µm PVDF filter exhibited 0% viability. This was true for both untreated and treated extracts, confirming the utility of a 0.1 µm, chemical-resistant, size exclusion filter. Indeed, repeated work with PVDF filters in lieu of cellulose filters by Weller *et al*. [7] demonstrated an almost complete lack of viable cells over 36 replicates, with one exception – a single colony found on a plate 7 days from a treated spore extract. What this means is uncertain, and the authors discuss considerations surrounding the risk of potential escape cells/spores.

It is possible that cells can ‘escape’ best practices in the community, and operational aspects must be considered as well. Filter integrity can be compromised through undue force; thus, the need for a centrifugation step to reduce the amount of possible intact cells or spores that are applied to the filter is recommended to reduce the possibility of a damaged filter. A failure rate of 1% has been reported in previous literature for 0.1 µm filters [14]. However, this was eliminated when centrifugal forces were reduced below 10,000 x g, presumably through limiting force on the filter material to cause tearing. Filter material treatment, storage, age, technical error in use, cross-contamination – one can expect that there is no process that is 100% risk free and use the evidence available to make informed decisions for their institution. Importantly, related work completed within our laboratory has demonstrated that the risk of exposure to viable cells falls to the operator during the extraction and spotting steps, and that swabbing within the mass spectrometer instrument after ionization of extracts containing viable cells did not result in any positive signal for the organism of interest [15]. Thus, the risk for exposure to operators within an institution remains within the processing steps that can be completed within a biosafety cabinet, inclusive of plate spotting and overlay.

Regardless, many facilities will continue to maintain and monitor high-risk extraction materials even with fulsome load testing data, with a zero-risk tolerance. This is particularly true for the release of materials external to the institution undertaking high-risk procedures, i.e. those that distribute control or proficiency material. With that perspective, questions remain regarding the optimal recovery media and time to detection for post-processing viability testing. It is reported that the recovery of viable cells is more effective with broth culture rather than direct plating [2,7]. Moreover, in the situation where a chemical extraction is taking place, the dilution/neutralization of the chemicals in the extract is also requisite to allow bacterial multiplication [4]. We were able to demonstrate within this study that broth culture did detect 100% of viable spore preparations compared to 83% using solid media, and that BHI seemed to have an advantage over SBA for detection purposes. While the numbers within this study are not exhaustive, it was not an unexpected finding. More relevant for operational risk assessments and standardization of operating protocols within a containment environment, the window for detection of viable, vegetative cells was up to 7 days, whereas the detection of viable spores could be detected as quickly as 1 day. This aligns with the findings from the CDC exposure in 2014, where viability plates were observed to have no growth at 24 h, and the direct plating of the extract may have contributed to inhibition [9]. Finally, the use of not-fit-for-purpose automated culture detection instruments, like the MGIT^©^ BACTEC 960 instrument, is not appropriate – the broth used within this system did signal growth of *B. anthracis* control material as expected, but did not signal growth within the treated samples, despite a 65 × dilution of the chemical (120 µl extract into 7.8 ml broth). Subsequent to the instrument’s 14-day monitoring period, the entire MGIT^©^ tube was plated to SBA and growth was confirmed, demonstrating that the small volume of chemical within the tube effectively inhibited bacterial growth within the broth, leading to a false assumption of non-viability. Taken together, this supports the appropriateness of a 14-day, combined enrichment broth and solid media viability method, with a neutralization step to mitigate inhibition. Overall, the data generated here provide evidence towards the development of community best practices and help inform institutional risk assessments for operations related to high-consequence bacterial pathogens, particularly those resistant to conventional inactivation methods.

## Abbreviations

BHI: brain heart infusion
CDC: Centre for Disease Control and Prevention
MALDI-TOF: matrix-assisted laser desorption/ionization-time of flight
PBS: phosphate buffered saline
qPCR: quantitative, real-time PCR
RG3: risk group 3
SBA: sheep blood agar
SEM: scanning electron microscope
TBS: tryptic soy broth
TFA: trifluoroacetic acid
